# Peripheral Deltorphin II Inhibits Nociceptors Following Nerve Injury

**DOI:** 10.3389/fphar.2020.01151

**Published:** 2020-07-31

**Authors:** Marek Joukal, Lucy Vulchanova, Cecilia Huffman, Petr Dubový, Christopher N. Honda

**Affiliations:** ^1^ Department of Anatomy, Cellular and Molecular Neurobiology Research Group, Faculty of Medicine, Masaryk University, Brno, Czechia; ^2^ Department of Neuroscience, University of Minnesota, Minneapolis, MN, United States

**Keywords:** nociceptors, spared nerve injury, delta opioid receptors, neuropathic pain, deltorphin II

## Abstract

Clinical and preclinical studies have revealed that local administration of opioid agonists into peripheral tissue attenuates inflammatory pain. However, few studies have examined whether peripherally restricted opioids are effective in reducing mechanical allodynia and hyperalgesia that usually follows nerve injury. The aim of the present study was to determine whether the mechanical responsiveness of C-fiber mechanical nociceptors innervating skin under neuropathic pain conditions is depressed by direct activation of delta opioid receptors (DORs) on their peripheral terminals. A murine model of peripheral neuropathic pain was induced with a spared nerve (tibial) injury, in which mice survived 7 or 28 days after surgery before electrophysiological testing began. Control groups comprised naïve and sham-operated animals. An ex vivo preparation of mouse plantar skin with attached tibial nerve was used to examine electrophysiologically the effects of the selective DOR agonist, deltorphin II, on the response properties of individual cutaneous C-fiber nociceptors. In contrast to naïve and sham-operated animals, deltorphin II induced an inhibition of the mechanical responsiveness of C-fiber mechanical nociceptors innervating skin under neuropathic conditions. The effects of deltorphin II were concentration-dependent and prevented by pretreatment with naltrindole indicating DOR-mediated inhibitory effects of deltorphin II. Our results provide the first direct evidence for expression of functional DORs on mechanical nociceptors innervating skin in an animal model of neuropathic pain.

## Introduction

Peripheral neuropathic pain, manifested as spontaneous pain, hyperalgesia, and allodynia, can result from many forms of nerve damage (Woolf and Mannion, 1999; Jensen et al., 2001). Current therapeutic approaches reduce, but do not eliminate, the hyperalgesia and allodynia. Systemically delivered opioids have very modest effects on neuropathic pain (Bian et al., 1999; Przewłocki and Przewłocka, 2001), usually requiring much higher doses for adequate relief (Portenoy and Hagen, 1990). Unfortunately, effective central analgesic actions of opioids are usually accompanied by untoward centrally mediated effects, such as sedation and respiratory depression, as well as peripheral effects, such as gastrointestinal disturbance and nausea. Moreover, prolonged opioid use can lead to tolerance and dependence. In view of the current opioid epidemic (Skolnick, 2018), there is an especially relevant need to seek out therapeutic alternatives to centrally acting opioids, such as manipulation of an endogenous peripheral opioid analgesia system (Stein, 2018). In the peripheral nervous system, opioid receptors are synthesized in somata of primary sensory neurons in dorsal root ganglia (DRG) then distributed centrally to axon terminals in superficial layers of spinal dorsal horn and peripherally to processes of small-caliber fibers (Coggeshall et al., 1997). During peripheral inflammation, synthesis of opioid receptors in DRG is upregulated, their axonal transport in peripheral nerves is enhanced, and peripheral density of receptors is elevated (Hassan et al., 1993; Mousa et al., 2001). Delta opioid receptors (DORs) have been immunohistochemically localized to axons innervating healthy skin (Coggeshall et al., 1997; Wenk and Honda, 1999), yet their functional competence under naïve conditions has been difficult to demonstrate.

It is well-established however, that peripherally restricted opioids are very effective under conditions of inflammation. Peripheral opioids attenuate behavioral hyperalgesia in models of inflammatory pain (Joris et al., 1987; Stein et al., 1988; Stein et al., 1989; Stein and Zöllner, 2009), and these effects are dose-dependent and antagonist-reversible (Ferreira and Nakamura, 1979; Levine and Taiwo, 1989; Joris et al., 1990; Barber and Gottschlich, 1992). In electrophysiological studies, locally applied morphine has been shown to inhibit spontaneous activity (Russell et al., 1987) as well as mechanical and thermal responses of nociceptors innervating inflamed skin while having no effects in healthy skin (Wenk et al., 2006). In addition, direct application of DOR agonists to receptive fields of nociceptors resulted in robust inhibition in inflamed, but not healthy skin (Brederson and Honda, 2015).

Less is known about the functional status or efficacy of peripheral opioid analgesic systems following nerve injury. In rodent models of neuropathic pain, peripheral delivery of mu opioid (Guan et al., 2008) and DOR agonists attenuates behavioral hyperalgesia (Obara et al., 2004; Kabli and Cahill, 2007; Obara et al., 2009). Kabli and Cahill (2007) also demonstrated concurrent increased expression of DORs in peripheral nerve and DRG following nerve injury. However, Obara et al. (2009) observed decreased levels of mRNA for DORs in spinal cord and DRG under similar conditions of nerve injury. In electrophysiological studies, peripheral delivery of mu opioid receptor agonists reduced the excitability of nociceptors following nerve injury (Schmidt et al., 2012). The existence of functional DORs on nociceptors under neuropathic conditions, and whether their activation contributes to attenuation of hyperalgesia and allodynia is unclear.

Therefore, the goal of the present study was to determine whether the mechanical responsiveness of nociceptors was attenuated by direct and localized activation of DORs on their peripheral terminals following spared tibial nerve injury (SNIt). Electrophysiological recordings in an isolated preparation of mouse hind paw skin with attached tibial nerve was used to examine effects of the selective DOR agonist, deltorphin II, on the responsiveness of C-fiber mechanical nociceptors innervating skin under experimental (SNIt) and control (naïve and sham-operated) conditions. We report that application of deltorphin II decreased evoked activity of skin nociceptors in the SNIt neuropathic pain model compared to control animals. This effect was prevented by co-application of naltrindole, a DOR-selective antagonist. These results provide direct evidence for existence of functional DORs on peripheral axon terminals of mechanical nociceptors innervating skin under conditions of neuropathic pain.

## Materials and Methods

### Animals and Surgical Procedures

All work with animals adhered to the guidelines of the Committee for Research and Ethical Issues of the International Association for the Study of Pain and was approved by the Institutional Animal Care and Use Committee at the University of Minnesota in accordance with American Veterinary Medical Association guidelines. Experiments were performed on 31 adult (25–35 g; 4-6 weeks) male outbred ICR/CD-1 mice (Envigo, Indianapolis, IN, USA). We induced spared nerve (tibial) injury (SNIt) as a model of peripheral nerve injury-induced neuropathic pain. The SNIt model of sparing the tibial nerve produces robust and consistent behavioral signs of neuropathic pain including reduction of nociceptive threshold (Shields et al., 2003). All surgical procedures were sterile and performed under deep isoflurane anesthesia. After incision of skin and muscle of the right hind limb, the sciatic nerve was exposed, and sural and common peroneal nerves were tightly ligated with 6-0 silk suture. Next, the ligated sciatic nerve branches were transected distal to the ligature, and approximately 2 mm of each distal nerve stump was removed. The retracted muscles were closed with absorbable suture (Ethicon) and the skin incision was closed with wound clips. In sham-operated animals, the right sciatic nerve was exposed, but no ligations or lesions were performed. The SNIt and sham-operated animals survived for 7 (sham, n = 4; SNIt, n = 8) and 28 days (sham, n = 4; SNIt, n = 9).

### Behavioral Mechanical Sensitivity

Mechanical sensitivity of the right hind paw (ipsilateral) was tested using Von Frey nylon monofilaments (Stoelting, Wood Dale, IL). Experimental and control animals were placed on a wire mesh grid under glass enclosures and allowed to acclimate for 30 min before behavioral testing. Tips of monofilaments were then pressed to the mid-plantar surface of the hind paw with enough bending force to cause the mouse to withdraw its paw from the tip, typically with a flinching behavior. Mechanical withdrawal thresholds were determined using the up-down method according to Chaplan et al. (1994) in sham-operated (n = 8) and SNIt mice (n = 8) 7 and 28 days after surgery. Data are reported as the mean percent of baseline withdrawal threshold ((postoperative threshold/baseline threshold) × 100) ± standard deviation (SD).

### Isolated Skin–Nerve Preparation

An isolated skin-nerve preparation (Reeh, 1986) was used for combined electrophysiological and pharmacological study of single afferent fibers innervating plantar skin of right (ipsilateral) hind paws. Experimental (SNIt) and control (naïve and sham-operated) animals were deeply anesthetized with isoflurane. The glabrous skin of the hind paw was dissected and excised together with the attached tibial nerve and the medial and lateral plantar nerves. The skin–nerve preparation was immediately transferred to a chamber continuously perfused (15–20 ml per minute) with warmed (26 ± 2°C) oxygen-saturated synthetic interstitial fluid [SIF; (Bretag, 1969)] containing (in mM) 123 NaCl; 3.5 KCl, 0.7 MgSO_4_, 2.0 CaCl_2_, 9.5 Na gluconate, 1.7 NaH_2_PO_4_, 5.5 glucose, 7.5 sucrose, 10.0 Hepes (pH 7.45 ± 0.05 mOsm, 290 ± 0.05 mOsm). Warmed and oxygenated SIF was used in all subsequent procedures. The preparation was then oriented corium side up and anchored with insect pins before being further dissected to clear the skin and nerve of all tendons, muscles, and vasculature. The cut end of the tibial nerve was threaded through a small aperture into an adjacent small recording chamber and placed on the surface of a mirrored dissection platform. The main chamber was continuously perfused with SIF, and the recording chamber was filled with SIF below, and oil above the mirror.

### General Electrophysiological Procedures

A compound action potential (neurogram) was recorded at the beginning of most experiments. A monopolar microelectrode (insulated except at tip) was placed on the main trunk of the medial or lateral plantar nerve for electrical stimulation. A large bundle of fibers was first divided from the main tibial nerve and lifted onto a fine gold wire electrode for extracellular recording. The recording electrode was suspended in the oil layer of the recording chamber and referenced to the bath with a silver/silver chloride electrode. Stimulating current was delivered with increasing intensity until each waveform component (Aαβ, Aδ, and C) of the compound action potential could be evoked and differentiated. The rate of conduction was calculated for each waveform and expressed as meters per second. Subsequently recorded single fibers were classified by conduction velocity based on the conduction velocities of the neurogram waveforms. When a particular waveform component could not be evoked for a given experiment, or if no compound action potential was recorded for an experiment, single units were classified according to the mean conduction rate of waveforms from compound action potentials recorded in all experiments. Electrical signals were differentially amplified (DAM50, World Precision Instruments, Austin, TX), filtered, and routed in parallel to an oscilloscope and computerized data acquisition system.

### Isolation of Single Units

Initially, small bundles of nerve fibers were teased from the nerve trunk and placed on the recording electrode to observe activity from multiple axons. The corium surface of the skin was then gently probed with a blunt glass rod to identify the general area of skin innervated by the small bundle. Next, electrical search stimuli were delivered to the nerve trunk through a microelectrode to elicit single fiber activity as progressively smaller filaments were isolated and placed on the recording electrode. Once single unit activity could be isolated, a second, roving, stimulating electrode was progressively traced along the plantar nerve branches until the receptive field could be electrically identified. Conduction velocity of individual axons was determined by electrical stimulation of the center of the receptive field and expressed as meters per second. Individual units were classified based on conduction velocity ranges obtained from whole-nerve compound action potential recordings made at the beginning of most experiments.

### Functional Characterization of Afferent Fibers

Because of sampling bias inherent in the search protocol (described above) and the limited ability to search with thermal stimuli, all afferent fibers encountered were mechanoreceptors. The mechanical threshold for each single unit was determined using a series of calibrated von Frey nylon monofilaments applied to the corium surface of the skin. Threshold was defined as the lowest bending force that consistently evoked an action potential response 50% of the time. Fibers were classified as nociceptors if they exhibited slowly adapting responses to sustained mechanical stimulation, and their firing rate increased monotonically with increasing force of stimulation. After functional characterization of each fiber, a small cylinder (5 mm diameter) was sealed over the receptive field with petroleum jelly and filled with SIF. The cylinder served as a reservoir for subsequent mechanical and thermal testing as well as drug delivery. The thermal responsiveness of fibers was qualitatively assessed by filling the cylinder sequentially with cold (5°C), warm, then hot (45°C) SIF. Thermal stimulation was used to complete the functional characterization of mechanical nociceptors, but changes in thermal responses were not evaluated during drug testing.

### Quantification of Mechanical Responses

After functional characterization, each afferent fiber was quantitatively tested for responses to mechanical stimulation before and after exposure to drug or vehicle. Mechanical stimulation was delivered by a von Frey filament with suprathreshold bending force that was mounted in a micromanipulator and lowered onto the receptive field encircled by the cylinder. Testing trials consisted of three 5-s periods of mechanical stimulation, each preceded by 10 seconds without stimulation. The response for each stimulation period was determined by subtracting the number of spikes in the preceding 5 seconds from each 5 second period of stimulation. The mean number of spikes of the three periods of stimulation represented the “response measure” for each trial. Data are recorded either as numbers of spikes (response measure) or spikes per second (Hz, firing rate). The stimulation onset and offset times were signaled to the online data acquisition program (see below).

### Spontaneous Activity

Un-evoked neuronal activity was recorded for at least 30 seconds before quantitative mechanical testing. Units were classified as having spontaneous activity if their firing rates were ≥ 0.1 Hz in the absence of any intentional stimulus.

### Preparation of Drugs

Stock solutions of deltorphin II (100 μM; Phoenix Pharmaceuticals, Belmont, CA) were made in water and stored at 4°C. Working concentrations of the ligand were diluted in SIF, as needed. Naltrindole hydrochloride (Tocris, Ellisville, MO) was reconstituted in water to a stock solution of 100 μM and was stored at −20°C. Each drug solution was warmed to room temperature and saturated with oxygen prior to use.

### Peripheral Delivery of Drug and Mechanical Testing

After functional characterization of each fiber, a small cylinder (5 mm diameter) was placed over the receptive field to serve as a reservoir for drug delivery. After the baseline (before drug) mechanical stimulation trial, the drug reservoir was emptied of SIF with a suction pipette and replaced with oxygenated deltorphin II, deltorphin II plus naltrindole, or vehicle (SIF) for 2 min. After 2 min of drug exposure, a second mechanical stimulus trial was performed before rinsing of cylinder with fresh SIF. Mechanical responses were retested every 15 to 30 min after drug washout to test for recovery. Recovery from drug application was defined as at least a 50% return towards baseline mechanical response. Units that did not recover were excluded from this study. Based on a previous electrophysiological study in inflamed skin (Brederson and Honda, 2015) and in preliminary spared nerve experiments, 300nM was used as the test concentration for deltorphin II. Concentration-response relationships were determined using cumulative ascending dosing without intervening rinses with SIF. Data are reported as the mean percent of baseline firing ((post-drug response/baseline response) × 100) ± standard deviation (SD) for each unit unless otherwise noted.

### Data Collection and Analysis

Compound action potentials, teased fiber recordings, and stimulus delivery times were collected with Spike 2 software and Power1401 interface (CED, Cambridge, England). Data analysis and spike discrimination were performed online and offline. STATISTICA 5.5 software (StatSoft, Tulsa, OK, USA) was used for statistical analysis and generation of graphs. Statistical analyses are described as necessary for each section of *Results*. Unless specified otherwise, values are expressed as means ± SD.

## Results

### Compound Action Potentials

Compound action potential recordings were made from the tibial nerve at the start of each experiment. Isolated single units were classified into fiber type based on comparison of their conduction velocities to those of compound action potential waveforms for that experiment. There was no difference in conduction velocities of compound action potential waveforms found between naïve, sham, and SNIt groups of animals. Combining all groups, the mean values for ranges of conduction velocities ( ± SD) of compound action potential waveforms were: Aαβ (n = 30) 22.7 to 10.5 m/s; Aδ (n =,29) 5.3 to 2.3 m/s; C (n = 29) 1.5 to 0.3 m/s.

### Functional Classification of Primary Afferent Units

A total of 42 single units isolated from the tibial nerves of 31 mice (experimental and control) were included in this study. All fibers conducted within the C-fiber range with a mean conduction velocity of 0.72 m/s ± 0.46. Receptive fields were evenly distributed across the plantar aspect of the foot, and no attempt was made to measure or otherwise quantify receptive field size. All fibers included in this study were functionally classified as nociceptors based on their stimulus–response relationships, and they were assigned to one of the following categories based on their response properties: C-mechanical nociceptors (CM), C-mechanoheat nociceptors (CMH), C-mechanocold nociceptors (CMC) and C-mechanoheat-cold nociceptors (CMHC) (**Table 1**).

**Table 1:** Distribution of functional categories of nociceptors among control and experimental groups.

	Naive	Sham-7D	Sham-28D	SNI-7D	SNI-28D
**CMN**	5 (83)	4 (80)	6 (60)	6 (46)	7 (78)
**CMH**	1 (17)	0	2 (20)	6 (54)	1 (11)
**CMHC**	0	1 (20)	0	0	1 (11)
**CMC**	0	0	2 (20)	0	0
**Total**	6	5	10	12	9

All units were classified as C fibers based on conduction velocities then classified based on response properties. C-mechanical nociceptors (CMN), C-mechanoheat nociceptors (CMH), C-mechanocold nociceptors (CMC) and C-mechanoheat and cold nociceptors (CMHC). Values are numbers of individual units with percentages of column totals in parentheses.

### Effects of Spared Nerve Tibial Injury on Mechanical Sensitivity

**Figure 1:**
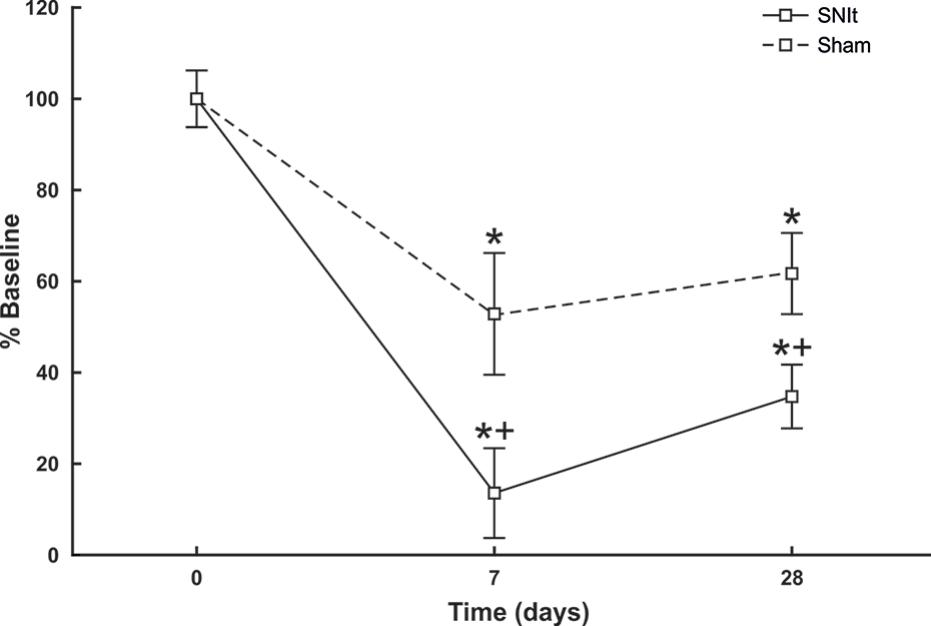
Behavioral assessment of SNIt model. Changes in withdrawal thresholds to mechanical stimulation were determined in sham-operated (n = 8) and SNIt mice (n = 8) 7 and 28 days after surgery. Sham surgery resulted in a significant decrease in withdrawal threshold at both survival times, and SNIt caused greater decreases in threshold compared to sham-operated animals. Changes in mechanical thresholds are reported as percent of pre-surgery baseline ± standard deviation of mean. *Significant difference when compared with baseline (Mann-Whitney U-test; P< 0.05). + indicates significant difference when compared with sham-operated animals (Mann-Whitney U-test; P< 0.05).

Behavioral testing revealed significant decreases in mechanical withdrawal thresholds for both SNIt and sham-operated animals at 7 and 28 days survival times, when compared to naïve animals. Moreover, the decrease in thresholds for SNIt animals was significantly greater than sham-operated animals at the same time points (P<0.05, Mann-Whitney *U* test) (**Figure 1**). **Table 2** summarizes comparisons of properties of individual nociceptors from control and nerve-injury groups. Conduction velocities and rates of spontaneous activity (axon firing in the absence of intentional stimulation) were not affected by sham or experimental nerve injury. Mechanical thresholds of individual fibers from sham-operated animals did not differ from those in the naïve group. However, after nerve injury, median mechanical thresholds of individual nociceptors in 7- and 28-day SNIt animals were significantly lower than both naïve and sham-operated animals (P<0.05, Kruskal-Wallis one-way ANOVA).

**Table 2:** Comparisons of properties of nociceptors in control and experimental groups.

	CV (m/s)	Spontaneous Activity (Hz)	Mechanical Threshold (mN)
**Naive**	0.63 ± 0.45	0.28 ± 0.12	10.35 (5.6, 23.1)
**Sham-7D**	0.63 ± 0.40	0.22 ± 0.18	10.00 (8.0, 26.9)
**Sham-28D**	0.75 ± 0.40	0.10 ± 0.15	14.55 (9.0, 23.1)
**SNIt-7D**	0.64 ± 0.51	0.18 ± 0.07	*6.15 (4.6, 13.4)
**SNIt-28D**	0.91 ± 0.54	0.15 ± 0.05	*4.08 (4.07, 10.0)

Conduction velocity (CV) and spontaneous activity (SA) are expressed as mean ± S.D., whereas mechanical thresholds (MT) are expressed as median and interquartile range. * indicates significant difference in mechanical threshold compared to fibers from naïve and corresponding sham groups (Kruskal-Wallis one way ANOVA, P < 0.05).

### Effects of Spared Nerve Tibial Injury on Sensitivity to Deltorphin II

**Figure 2:**
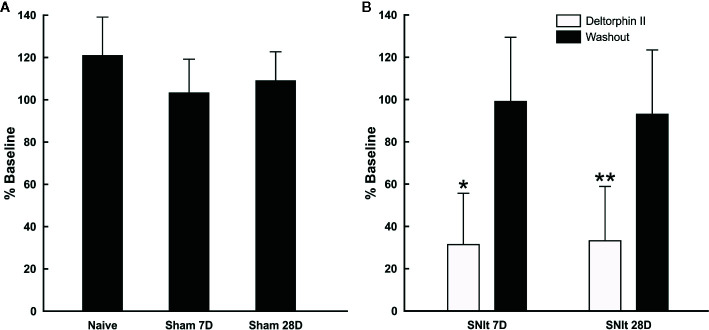
Responses of afferent fibers from control and nerve-injured animals after exposure to deltorphin II. **(A)** Deltorphin II did not reduce responses of nociceptors to mechanical stimulation in naïve or sham-operated animals (P > 0.05, Mann-Whitney U-test). Response measures for each condition are presented as the mean percent of baseline response ( ± SD) to mechanical stimulation after application of 300 nM deltorphin II in naïve (120.9 ± 18.2; n = 5) and sham-operated animals surviving 7 days (103.2 ± 16.0; n = 6) and 28 days (108.9 ± 13.7; n = 10) after surgery. **(B)** Deltorphin II reduced responses of afferent fibers to mechanical stimulation after peripheral nerve injury (*P < 0.05, **P < 0.01; Mann-Whitney U-test). Response measures for each condition are presented as the mean percent baseline response (± SD) to mechanical stimulation after application of 300 nM deltorphin II in SNIt operated mice surviving 7 days (31.92 ± 24.46; n = 12) and 28 days (33.52 ± 25.84; n = 9) after surgery.

**Figure 3:**
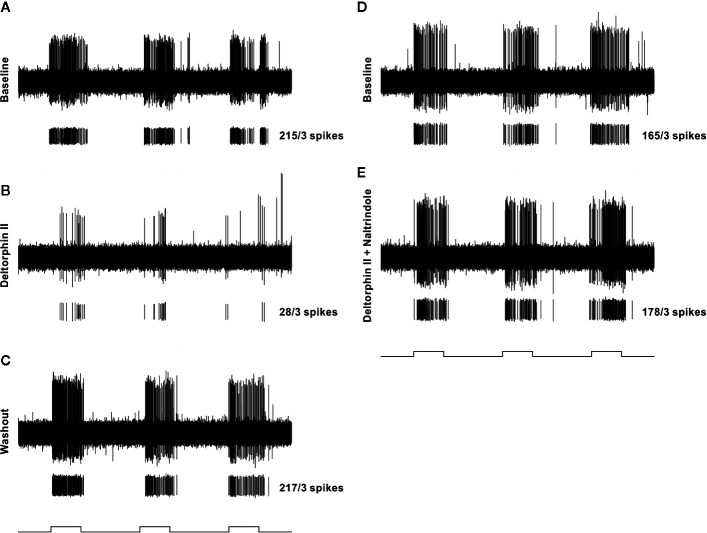
Representative example of testing protocol and responses of an individual nociceptor before and after localized administration of test drugs that were restricted to a cylinder centered over the receptive field. In each panel **(A–E)**, the top trace is a raw recording from a C-fiber mechanical nociceptor (CV 0.61 m/s; mechanical threshold 4.1 mN). Each lower trace contains individual spikes sorted in software from the raw trace along with the total number of spikes counted during the three stimulation periods. The test mechanical stimulus was 38 mN, and stimulus timing (5 s, each separated by 10 secs) is indicated at the bottom of each column. The left column shows baseline response before drug **(A)**, response 2 min after 300 nM deltorphin II **(B)**, and response after 20 min of washout **(C)**. After washout, a new baseline was established **(D)** before application of 300 nM deltorphin II plus 300 nM naltrindole **(E)**.

Sensitivity of individual nociceptors to deltorphin II was tested in experimental and control animals. **Figure 2A** illustrates the lack of effect of deltorphin II in control animals on the responses of individual fibers to mechanical stimulation after drug application. The mean percent baseline responses after drug application in naïve animals (120.9 ± 18.2) and in sham operated animals 7 days (103.2 ± 16.0) and 28 days (108.9 ± 13.7) after surgery were not significantly different (P > 0.05, Mann-Whitney *U* test). In contrast, peripheral application of deltorphin II produced robust inhibition of responses to noxious mechanical stimulation in nociceptors of SNIt-operated mice (**Figure 2B**). The mean percent baseline response following 300 nM deltorphin II in SNIt operated mice was 31.9 ± 24.5% after 7 days and 33.5 ± 2.8% after 28 days. Fibers recovered from inhibition in less than 1 h (range, 15–60 h) after drug rinse and washout. A representative example of the effect of deltorphin II on an individual nociceptor from a 28 days SNIt animal is shown in **Figure 3**.

### Deltorphin II Concentration-Response Relationship

**Figure 4:**
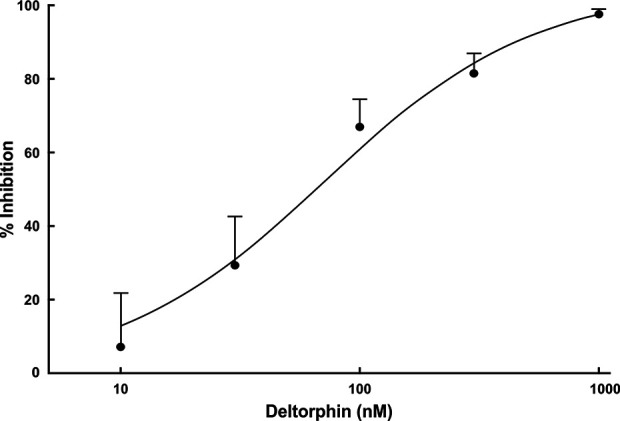
Deltorphin II concentration-response relationship. Sequential application of deltorphin II (10, 30, 100, 300, 1,000 nM) directly to the receptive fields of individual C-fiber nociceptors (n = 4) innervating skin in SNIt animals 28 days after surgery reduced responses to mechanical stimulus (38 mN) in a concentration-dependent manner. Data points represent mean percent inhibition of response in presence of drug, relative to baseline response of each unit ( ± SEM). Estimated EC_50_ value is 73.5 nM. Regression lines and estimated EC_50_ value were calculated using STATISTICA 5.5.

Concentration response relationships were determined in a subset (n = 4) of nociceptors (SNI 28 days) that all responded with inhibition to 300 nM concentration of deltorphin II. After recovery from the initial 300 nM test, a series of increasing concentrations of deltorphin II (10, 30, 100, 300, and 1,000 nM) was applied to receptive field of the same fiber for 2 min each. A baseline mechanical stimulus trial was first performed with fresh SIF. The SIF was next replaced with 10 nM deltorphin II solution for 2 min before a second mechanical stimulus trial was performed in the presence of deltorphin II. The reservoir was then emptied, and the next higher concentration of drug was added, and after a 2-min incubation, another mechanical stimulus trial was performed. This protocol was repeated for the remaining concentrations of deltorphin II before the drug was washed out and recovery was tested. All fibers included in construction of the dose response curve recovered in 15 to 35 min. Mechanical responses were expressed as percent of the initial baseline trial response. Percent response inhibition was calculated, and a concentration response curve was constructed (**Figure 4**).

### Inhibitory Effects of Deltorphin II Are Prevented by Naltrindole

**Figure 5:**
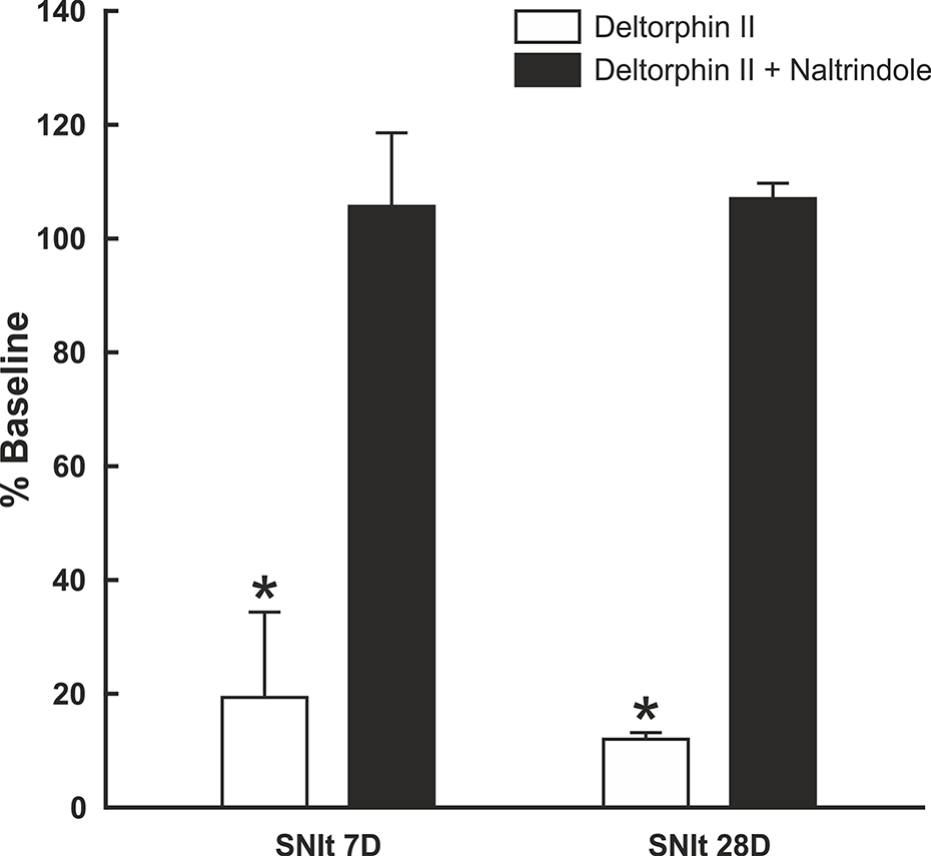
Inhibitory effects of deltorphin II were prevented by naltrindole. Nociceptors from mice 7 days (n = 3) and 28 days (n = 2) after SNIt were first tested with 300 nM deltorphin II. After washout and recovery, they were re-tested with equimolar (300 nM) concentrations of deltorphin II plus naltrindole. Mean percent baseline response ( ± SD) was decreased to 19.4 ± 15.1% (SNIt 7D) and 12.0 ± 1.2% (SNIt 28D) when deltorphin II was applied alone, compared to 105.7 ± 12.9% (SNIt 7D) and 107.0 ± 2.7% (SNIt 28D) of baseline when deltorphin II was co-applied with naltrindole. *Significant difference compared to baseline (P < 0.05, paired t-test).

To test whether the inhibitory effects of deltorphin II were mediated by DOR, equimolar concentrations of deltorphin II (300 nM) and the selective DOR antagonist, naltrindole (300 nM), were co-applied to nociceptors 7 days (n = 3) and 28 days (n = 2) after SNIt surgery. Responses to mechanical stimulation were first inhibited by deltorphin II alone to 19.4 ± 15.1% of baseline at 7 days and to 12.0 ± 1.2% of baseline at 28 days after SNIt (P<0.05, paired t-test). Following washout and recovery from deltorphin II (alone) a new baseline mechanical trial was performed for each nociceptor before co-delivery of deltorphin II and naltrindole. **Figure 5** shows that naltridole reversed the inhibitory effects of deltorphin II at 7 days (105.7 ± 12.9% of baseline) and 28 days (107.0 ± 2.7% baseline) after SNIt (P > 0.05, paired t-test).

## Discussion

The objective of the present study was to determine if functional DORs are expressed on the peripheral processes of cutaneous nociceptors in a rodent model of neuropathic pain resulting from a nerve injury with spared tibial nerve (SNIt). We tested this idea by assessing responses to mechanical stimulation in the presence of deltorphin II administered locally in naive, sham- and SNIt-operated mice. Application of deltorphin II directly to the receptive fields of afferent fibers innervating skin of SNIt animals suppressed responses to noxious mechanical stimulation in all units studied. Deltorphin II effects were concentration-dependent and prevented by pretreatment with naltrindole indicating that deltorphin II effects were DOR-mediated. These data provide direct electrophysiological evidence for functional DORs on the peripheral terminals of somatic afferent neurons under neuropathic conditions.

### Spared (Tibial) Nerve Injury Induced Behavioral Allodynia and a Corresponding Decrease in Thresholds of Mechanical Nociceptors

Spared nerve injury (SNI) is a model of partial-limb denervation with minimal variability of nerve damage. The plantar area of the hindlimb is innervated by three branches of the sciatic nerve, the common fibular, the tibial, and the sural nerves (Swett and Woolf, 1985). In contrast to other models of peripheral nerve injury, SNI models produce a consistent degree of axonal damage. Even if only two nerve branches out of three are axotomized, there will be changes in soma found in all DRGs associated with the sciatic nerve. Moreover, there is considerable co-mingling of cell bodies of injured and uninjured neurons in corresponding DRG (Klusáková and Dubový, 2009). The classic SNI model described by Decosterd and Woolf (2000) was based on transection of the tibial and the common fibular nerves, leaving the sural nerve intact.

In the present experiments we utilized a modification of SNI, in which the tibial nerve was left uninjured. The SNIt model in mice produces robust behavioral mechanical allodynia accompanied by consistent anatomic changes in IB4-binding afferent terminals in the spinal cord (Shields et al., 2003). Wallerian degeneration causes electrophysiological changes in injured nerve axons (Chaudhry and Cornblath, 1992), so an important feature of the SNIt model is that it permitted recording from uninjured fibers some distance from degenerating axons. The present behavioral tests showed clearly that SNIt caused significant decreases of mechanical withdrawal threshold 7 and 28 days after injury compared to sham-operated animals. However, sham-operated animals also displayed a smaller decrease in mechanical withdrawal thresholds that persisted at 28 days. It is unclear why the results from the present behavioral studies do not agree completely with results from other laboratories. It is possible that there were unknown issues in the group of behavioral animals related to testing protocols or surgical technique. It is important to note that the decrease in behavioral mechanical sensitivity was not paralleled by a decreased mechanical sensitivity in individual nociceptors isolated from sham-operated animals. Importantly, electrophysiological study of tibial nerve afferent axons in SNIt-operated animals at the same time points revealed a significant decrease in mechanical threshold of nociceptors. Our observation of a lowering of mechanical threshold in nociceptors following spared tibial nerve injury that is not accompanied by changes in spontaneous activity is similar to reports from a study of spared sural nerve injury (Smith et al., 2013). We were not able to test consistently for the electrophysiological correlate of behavioral hyperalgesia (increased neuronal activity in response to identical noxious stimulation). Nor, did we detect a change in the incidence or rate of spontaneous activity following SNIt. The SNIt model of neuropathic pain has proven particularly valuable for combined electrophysiological and behavioral study following nerve injury because the tibial nerve is not directly involved with the nerve injury, and the innervation territory of the tibial nerve on the plantar surface of the hindlimb is amenable to behavioral testing.

### Deltorphin II Inhibits Responses of C-Fiber Nociceptors to Mechanical Stimulation After Nerve Injury

Many earlier studies evaluating effects of opioid receptor agonists in neuropathic pain models were based on their systemic administration. However, fewer studies have examined the effects of peripherally restricted opioids. Experiments of local administration of morphine, [D-Ala2, N-MePhe4, Gly-ol]-enkephalin – DAMGO, endomorphine 1, endomorphine 2, and peripheral κ-opioid receptor agonist asimadoline revealed anti-allodynic and anti-hyperalgesic effects in neuropathic pain models (Walker et al., 1999; Pertovaara and Wei, 2001; Truong et al., 2003; Obara et al., 2004). It has also been shown that peripherally restricted opioids, in the form of small molecules, are effective under similar conditions (Obara et al., 2007).

The present results demonstrate that nerve injury induces the expression of functional DOR on skin nociceptors. Responses to mechanical stimulation of all studied nociceptors in SNIt-operated animals was decreased by more than 60% from baseline after deltorphin II application. Deltorphin II effects on C mechanical nociceptors were dose-dependent and prevented by application of naltrindole, providing direct pharmacological evidence that the inhibitory effects of deltorphin II were DOR-mediated. Our results are in agreement with those of Kabli and Cahill (2007) who described effects of deltorphin II intraplantar injection on attenuation of behavioral hyperalgesia and mechanical allodynia after chronic constriction injury.

### Functional DOR Are Present on C Fibers Under Neuropathic Conditions, but Not in Naive or Sham-Operated Animals

Few studies have focused on changes of DOR in the peripheral nervous system following nerve injury. Some have shown that DOR expression decreased in corresponding DRG neurons after nerve injury (Zhang et al., 1997; Obara et al., 2009). However, Kabli and Cahill (2007) found up-regulation of DOR in DRG neurons after chronic constriction injury. Nevertheless, we found that nociceptors are very sensitive to DOR agonists 7 and 28 days after nerve injury, whereas nociceptors from naive and sham-operated animals do not appear to express functional DOR.

It is unclear how DOR become functionally competent on C-fiber nociceptors. One possibility is that partial nerve injury induces an increase density and sprouting of uninjured axons in their native uninjured dermatomes, i.e. in the skin area showing allodynia (Duraku et al., 2012; Duraku et al., 2013; Kuner and Flor, 2017). Nerve sprouts of regenerating C-fibers develop an early chemosensitivity to various substances including, e.g. bradykinin, histamine, serotonin, and capsaicin (Leah et al., 1988; Zimmermann, 2001). Another possible mechanism could be the paracrine secretion of proinflammatory cytokines and chemokines from injured neurons (Scholz and Woolf, 2007; Bai et al., 2016) that might evoke changes in expression of functional DOR in non-injured neurons. Finally, it is possible that a class of DOR-sensitive mechanically insensitive afferent (MIA) C-fibers becomes activated by tissue injury, inflammation or nerve injury (Meyer et al., 1991). Since MIA form a substantial proportion of the nociceptor population, the acquisition of mechanosensitivity after nerve injury may determine their role in development and maintenance of hyperalgesic and hypersensitive states (Gold and Gebhart, 2010).

The current findings provide new insight into intrinsic peripheral mechanisms of opioid analgesia that become enabled or engaged following damage to the peripheral nervous system. They highlight the importance of development of novel exogenous opioid agonists that can be delivered at low concentrations directly to peripheral targets or of agonists that can be delivered systemically but lack access to the central nervous system. Moreover, these findings suggest the more general need to develop therapeutic strategies that take advantage of an existing peripheral analgesic system including manipulation of levels of functional opioid receptors on peripheral afferent terminals, recruitment of peripheral opioid-producing cells, and synthesis and release of endogenous opioids.

## Conclusions

The results of this study provide direct evidence for the expression of functionally competent DOR on the peripheral processes of nociceptors innervating skin under neuropathic conditions. We have shown that the selective DOR agonist deltorphin II decreased responsivity of cutaneous nociceptors to mechanical stimulation after nerve injury. In contrast, mechanical nociceptors innervating normal skin are not sensitive to deltorphin II, and they do not appear to express functional DORs. Furthermore, our data suggest potential clinical utility of peripherally restricted DOR agonists for the treatment of neuropathic pain.

## Data Availability Statement

The raw data supporting the conclusions of this article will be made available by the authors, without undue reservation.

## Ethics Statement

The animal study was reviewed and approved by Institutional Animal Care and Use Committee at the University of Minnesota.

## Author Contributions

MJ: Conceptualization, investigation, writing-original/review, funding. LV: supervision, methodology, writing-review/edit, funding. CH: Investigation. PD: Conceptualization, writing-review. CNH: Conceptualization, methodology, supervision, writing-review/edit, funding.

## Funding

This work was supported by PHS grants DA09641 (CNH), DE021996 (LV), NS088518 (LV), Proshek-Fulbright Scholarship (MJ) and funds from the Faculty of Medicine, Masaryk University to junior researcher Marek Joukal grant No. ROZV/24/LF18/2018 and ROZV/23/LF14/2019.

## Conflict of Interest

The authors declare that the research was conducted in the absence of any commercial or financial relationships that could be construed as a potential conflict of interest.
